# Molecular engineering of a spheroid-penetrating phage nanovector for photodynamic treatment of colon cancer cells

**DOI:** 10.1007/s00018-024-05174-7

**Published:** 2024-03-17

**Authors:** Eleonora Turrini, Luca Ulfo, Paolo Emidio Costantini, Roberto Saporetti, Matteo Di Giosia, Michela Nigro, Annapaola Petrosino, Lucia Pappagallo, Alena Kaltenbrunner, Andrea Cantelli, Valentina Pellicioni, Elena Catanzaro, Carmela Fimognari, Matteo Calvaresi, Alberto Danielli

**Affiliations:** 1 Dipartimento di Scienze per la Qualità della Vita (QUVI), Alma Mater Studiorum, Università Di Bologna, C.So D’Augusto, 237, 47921 Rimini, Italy; 2 Dipartimento di Farmacia e Biotecnologie (FaBiT), Alma Mater Studiorum, Università Di Bologna, Via Francesco Selmi 3, 40126 Bologna, Italy; 3 Dipartimento di Chimica “Giacomo Ciamician”, Alma Mater Studiorum, Università Di Bologna, Via Francesco Selmi 2, 40126 Bologna, Italy; 4 CNR Institute of Molecular Genetics “Luigi Luca Cavalli-Sforza” Unit of Bologna, Bologna, Italy; 5 Cell Death Investigation and Therapy (CDIT) Laboratory, Department of Human Structure and Repair, Ghent University, Corneel Heymanslaan 10, 9000 Ghent, Belgium; 6 Cancer Research Institute Ghent (CRIG), Ghent, Belgium; 7 Interdepartmental Center for Industrial Research (CIRI-SDV), Health Sciences and Technologies, University of Bologna, Bologna, Italy

**Keywords:** PDT, Colorectal cancer, M13, Bacteriophage, Spheroids, Nanovector

## Abstract

Photodynamic therapy (PDT) represents an emerging strategy to treat various malignancies, including colorectal cancer (CC), the third most common cancer type. This work presents an engineered M13 phage retargeted towards CC cells through pentavalent display of a disulfide-constrained peptide nonamer. The M13_CC_ nanovector was conjugated with the photosensitizer Rose Bengal (RB), and the photodynamic anticancer effects of the resulting M13_CC_-RB bioconjugate were investigated on CC cells. We show that upon irradiation M13_CC_-RB is able to impair CC cell viability, and that this effect depends on i) photosensitizer concentration and ii) targeting efficiency towards CC cell lines, proving the specificity of the vector compared to unmodified M13 phage. We also demonstrate that M13_CC_-RB enhances generation and intracellular accumulation of reactive oxygen species (ROS) triggering CC cell death. To further investigate the anticancer potential of M13_CC_-RB, we performed PDT experiments on 3D CC spheroids, proving, for the first time, the ability of engineered M13 phage conjugates to deeply penetrate multicellular spheroids. Moreover, significant photodynamic effects, including spheroid disruption and cytotoxicity, were readily triggered at picomolar concentrations of the phage vector. Taken together, our results promote engineered M13 phages as promising nanovector platform for targeted photosensitization, paving the way to novel adjuvant approaches to fight CC malignancies.

**Supplementary Information:**

The online version contains supplementary material available at 10.1007/s00018-024-05174-7.

## Introduction

Colorectal cancer (CC) is the third most common cancer type worldwide and represents the second cause of cancer deaths. In 2020, almost 2 million cases were diagnosed, and the International Agency for Research on Cancer (IARC) estimates that the global burden of CC will increase by 56% between 2020 and 2040, reaching more than 3 million new cases per year [1]. Depending on the stage of cancer, chemotherapy, radiation therapy, target therapy and surgery are the current treatment modalities. However, regardless of diagnostic and therapeutic advances, tumor recurrence and metastasis are two critical factors that affect the survival rates of patients with CC [2].

Photodynamic therapy (PDT) is a minimally invasive strategy emerging as a valuable therapeutic procedure for the management of a variety of solid tumors [3, 4]. Despite being known since early 1900, its clinical applications are more recent [5]. PDT is currently being investigated as a treatment modality for CC and could be incorporated into neoadjuvant treatment plans to control primary and metastatic CC tumor growth by eliminating CC stem cells [2, 6–8]. PDT involves the use and uptake of non-toxic photosensitizers, which should preferentially accumulate in target tumor tissue. The photosensitizer (PS) is selectively activated by a light source whose emission spectra must overlap the absorption band of the PS. In presence of in situ oxygen (O_2_), the activated PS produces reactive oxygen species (ROS) that provoke oxidative stress and lead to cancer cell death [9]. The photoinduced oxidative stress mainly damages membranes and proteins. Photosensitizers that display a higher degree of accumulation inside and/or towards specific cells and organelle membranes are usually more cytotoxic and selective than free circulating photosensitizers [10]. Together with the direct damage of cancer cells induced by ROS generation, PDT can hamper cancer progression through the inhibition of neoangiogenesis and can stimulate the patient’s immune system by increasing cancer cell-derived antigen presentation to T cells, improving its therapeutic potential [5, 11, 12].

Since the main biological effects of PDT are limited by tissue accessibility of various light irradiation sources (lamps, lasers, optical fibers), its clinical application as anticancer strategy is mainly constrained to superficial or surgical-accessible regions. Moreover, the low cellular uptake of photosensitizers or their weak specificity for tumor tissues represent some of the main shortcomings affecting PDT efficacy [13, 14]. Within non-targeted drug delivery mechanisms, only minor amounts of PS passively accumulate in tumor sites and the remainder distributes into healthy tissues, causing unwanted side effects. Thus, specific targeting for tumors, combined with the spatial control of light delivery, would significantly contribute to minimize side effects of PDT [15].

Bacteriophages (phages) are viruses that only infect bacteria. The possibility to display tumor-targeting moieties on the capsid shell together with the innate capability of phages to penetrate tissues and barriers makes them ideal vectors to target cancer and tumor-associated cells [16–19]. Indeed, they are emerging as safe nanocarrier platforms to specifically direct PS to cancer cells [20]. In particular, the M13 phage has been recently shown to represent a very promising vector platform to target specifically epidermal growth factor (EGFR)-overexpressing cancer cells in PDT, given the high payload of conjugable PS on the capsid shell and the relative ease of genetic manipulation provided by well-established phage-display techniques [21, 22].

The present study employs an engineered M13 phage displaying a peptide nonamer shown to specifically target CC cells [23, 24]. Rose Bengal (RB) is a fluorescent dye approved by the Food and Drug Administration (FDA) as ocular diagnostic stain and orphan drug to treat certain cancers [25]. Since RB is endowed with high performances as light-induced ROS generator [26] it was conjugated to the phage capsid. We investigated the photodynamic anticancer potential of the resulting M13_CC_-RB bioconjugate. In particular, we characterized the tropism towards CC cell lines and the cytotoxic activity upon light irradiation. Finally, to widely explore the anticancer PDT potential, we carried out experiments in 3D CC models to verify the translational features of the phage vector platform, including penetration and lethal photosensitization of CC spheroids.

## Materials and methods

### Phagemid cloning and phage production

**Fig. 1:**
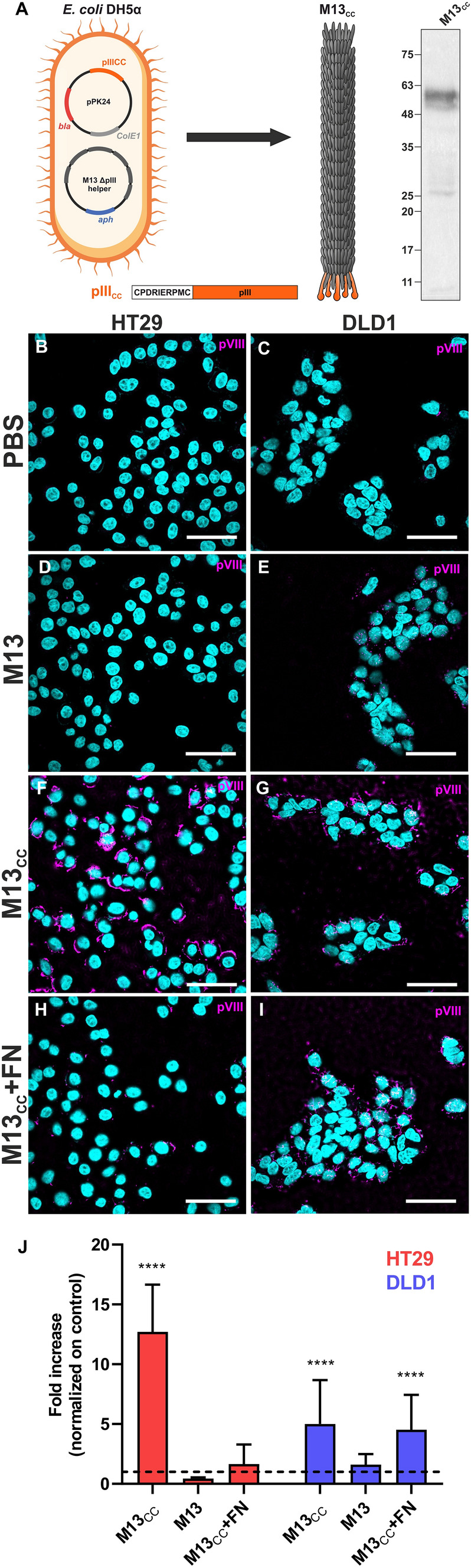
M13_CC_ targets CC cells.** A** Phage modification scheme, The CPDIRIERPMC coding sequence was cloned in frame with pIII (orange) generating the pPK24 plasmid. Transformed bacteria superinfected with Hyperphage helper produce modified phages. Immunoblot of M13_CC_ demonstrates incorporation of modified pIII in the purified virions. Immunohistochemical confocal microscopy of HT29 (**B**, **D, F, H**) and DLD1 (**C, E, G, I**) cell lines incubated with PBS (**B**, **C**), M13 (**D, E**), M13_CC_ (**F, G**) and M13_CC_ after preincubation with fibronectin (FN) (**H**, **I**). Nuclei are in cyan while the major coating protein pVIII of phage is in magenta. Scale bar = 50 µm. **J** Quantitative analysis performed on confocal images. Fluorescence intensity detected in confocal images were expressed as fold increase in comparison to the control PBS (dashed line) (n = 20). Statistical significance was calculated by one-way parametric ANOVA in comparison to the control (PBS); ****p < 0.0001

The M13 phage (M13K07 New England Biolabs, Ipswich, MA, USA) has been genetically modified to display a specific targeting peptide, CPDIERPMC, fused in frame with the pIII capsid protein [23]. The peptide coding sequence (CDS) was obtained by annealing oligonucleotides AD0186 (CATGGCCTGTCCAATTGAAGATAGGCCTATGTGTGGTGGCGGTG) and AD0187 (GATCCACCGCCACCACACATAGGCCTATCTTCAATTGGACAGGC) generating NcoI and BamHI overhangs. This insert was directionally cloned into the pSex81 vector (ProGen, Heidelberg, Germany) linearized with the same restriction enzymes (NEB, New England Biolabs). The resulting phagemid (pPK24), carrying the CPDIERPMC peptide fused in frame with the pIII CDS, preceded by a PelB leader sequence allowing for periplasmatic localization and virion incorporation of the fusion protein (Fig. 1I), was validated by Sanger sequencing and Immunoblotting for expression of the fusion construct. After transformation in *E. coli* TG1, positive colonies were selected by ampicillin resistance and superinfected with Hyperphage helper phage (ProGen), to achieve pentavalent type 3 display of the pIII peptide fusion. Infected bacteria were grown overnight in LB medium supplemented with ampicillin (100 mg/L), kanamycin (25 mg/L) and 0.4 mM isopropyl ß-D-1-thiogalactopyranoside (IPTG). IPTG was added to induce the expression of the CPDIERPMC-pIII fusion, ampicillin was added to select pPK24-positive bacteria, and kanamycin was added to select only Hyperphage-superinfected bacteria. After growth, culture was pelleted for 30 min at 6000*g* to remove bacteria, the supernatant with the M13_CC_ virions was collected and supplemented with 4% w/v of polyethylene glycol (PEG) 8000 and 3% w/v NaCl. The solution was incubated for 90 min at 4° C and then centrifugated for 15 min at 15000*g* at 4 °C. Pelleted phages were resuspended in sterile phosphate buffered saline (PBS) 1×. Phage concentrations were calculated by measuring the absorbance at 269 nm in a UV–Vis spectrophotometer (Agilent Cary-60, Agilent, Santa Clara, CA, USA) using an extinction coefficient of ε = 3.84 cm^2^ mg^−1^. The wild type phages (M13) were produced starting from un-transformed TG1 colonies grown to OD_600nm_ = 0.4 and infected with M13K07 Helper phage (NEB), culture was growth overnight in kanamycin supplemented LB for the selection of M13K07-infected bacteria. Purification was performed as described before.

### Immunoblotting

M13_CC_ phages at a concentration of 10^10 pfu/µL were resolved on a 12% SDS–polyacrylamide gel. Following electrophoresis, the proteins were transferred onto a PVDF (polyvinylidene difluoride) membrane (Immobilion-P, Millipore, France). The membrane was then blocked with a Blocking Solution (1X PBS pH 7.4, 5% milk, and 0.05% Tween), and then incubated with Anti-M13 pIII Monoclonal Antibody (New England BioLabs, Ipswich, MA, USA) diluted 1:5000 in the blocking solution for 1 h at room temperature. After this incubation, the membrane was then washed three times with Washing Solution (1X PBS and 0.05% Tween), and further incubated with a horseradish peroxidase (HRP)-conjugated IgG anti-mouse secondary antibody (Jackson ImmunoResearch Laboratories, West Grove, PA, USA) diluted 1:10,000 for 1 h at room temperature. The membrane was developed using an enhanced chemiluminescence (ECL) solution, comprising 1.25 mM luminol in 100 mM Tris (pH 8.8), 6.8 mM coumaric acid, and 30% hydrogen peroxide. Chemiluminescent signals were captured using the ChemiDoc™ Imaging System (Bio-Rad, Basel, Switzerland).

### Phage vector conjugation with the RB photosensitizer

The RB carboxylic group was covalently conjugated to the free amine-containing residues on the capsid of M13 and M13_CC_ via cross-coupling reaction using 1-ethyl-3-(3-dimethylaminopropyl) carbodiimide (EDC)/N-hydroxysuccinimide (NHS), resulting in the formation of amide bonds. NHS and EDC were added under stirring to a 10 mM RB solution in DMSO, reaching final concentrations of 15 mM NHS and 10 mM EDC. The mixture was incubated in dark conditions for three hours at 25 °C under constant shaking at 700 rpm. Then, 50 μL of the activated RB solution were added dropwise to 1 mL of PBS solution containing 40 nM of phages (M13 or M13_CC_). After the addition, each solution was incubated overnight in dark conditions at 25 °C, under shaking at 700 rpm. To remove unreacted RB and other reaction byproducts, the bioconjugates were dialyzed (14 kDa cut-off regenerated cellulose membrane) *versus* 100 mM sodium carbonate buffer at pH 9. The last dialysis step was carried out *versus* PBS.

### Cell cultures

Authenticated CC cell lines HT29 (mismatch repair [MMR] proficient cells), DLD1 and HCT116 (MMR deficient cells), as well as non-CC cell line A431 and BT474, were purchased from American Type Culture Collection (ATCC, Manassas, VA, USA). HT29 and HCT116 were cultured in McCoy’s 5A medium, while DLD1, A431 and BT474 in RPMI 1640. Both media were supplemented with 10% heat inactivated fetal bovine serum (FBS), 1% l-glutamine 200 mM and 1% penicillin/streptomycin solution 100 U/mL (all purchased from Euroclone, Pero, Italy). Cells were cultured at 37 °C in a 5% CO_2_ humidified incubator.

### Spheroid generation

To reproducibly generate multicellular spheroids of uniform size, we cultured CC cells using the low adhesion multiwell plate. Spheroids were formed using 96U Bottom Plate (Nunclon Sphera, Thermo Fisher Scientific, Waltham, MA USA). Briefly, 500 cells were seeded as 100 μL per well by centrifugation. After seeding, plates were cultured at 37 °C with 5% CO_2_ for 5 days to allow cell assembly and the formation of the spheroids. Half of the culture medium was replaced every three days. All steps were performed using an automatic multichannel pipette at a flow rate of 10 μL/s (Gilson, Middleton, WI, USA). Following microtissue formation, spheroids were transferred in the GravityTrap recipient plate (Insphero AG, Schlieren, Switzerland) allowing monitoring of the spheroid growth, viability, and microscope analysis. Spheroids displayed a slow, exponential, and homogeneous growth that we monitored for 5 days. According to literature data [27], proliferating cells were mainly detectable in the ring of cells localized in the outer layer of the spheroid, while the inner core was characterized by live quiescent cells or dying cells.

### Cell viability assay and analysis of cell death mechanisms

Cells were treated and incubated in complete medium with increasing PS concentrations (0.01 to 1 µM) of RB alone or M13_CC_-RB, corresponding to picomolar concentrations of bacteriophage, for 45 min. After incubation, cells were washed twice with PBS 1X, to remove the excess of bioconjugated phages.

Cells were irradiated for 30 min or shorter time according to experimental exigencies in PBS with a low irradiance white light LED (24 mW/cm^2^). These conditions were determined by comparing the efficacy of several irradiation times (from 10 to 30 min). No significant differences were observed at the highest tested concentrations of RB equivalents (1 µM). However, a significant difference was observed at 0.1 µM, where we recorded a higher percentage of cytotoxic effects for M13_CC_-RB after 30 min exposure compared to 10 min (data not shown). For this reason, 30 min light exposure was used as experimental condition for all the following experiments, unless differently reported. In parallel, cells were treated according to the before mentioned conditions and kept in the dark. After irradiation or dark incubation, cells were recovered for 24 h or shorter in complete medium. The analysis of cell viability was performed using the metabolic MTT (3-(4,5-dimethylthiazol-2-yl)-2,5-diphenyltetrazolium bromide) assay (Merck, Darmstadt, Germany) for both irradiated and not irradiated cells, as previously described [22].

Cell viability after PDT with M13_CC_-RB in presence of ROS inhibitor was performed as described above with few modifications. During incubation of cells with M13_CC_-RB, irradiation and recovery after treatment, media were supplemented with either N-acetyl-l-cysteine (NAC) or vitamin E at the final concentration of 10 mM and 100 µM, respectively.

To investigate the mechanism of cell death induced by M13_CC_-RB, after 3, 6 or 24 h from treatment, cells were washed, gently trypsinized using TrypLE (Gibco, Thermo Fisher) and incubated with 100 µL of Guava Nexin Reagent (Luminex, Austin, Texas, USA), containing 7-aminoactinomycin (AAD) and annexin V-phycoerythrin (PE), for 20 min at room temperature in the dark. After incubation, cells were analyzed using the flow cytometer Guava EasyCyte 6-2L (Luminex). Considering that at the highest concentration of M13_CC_-RB tested we recorded a signal spill over into the PE fluorescence channel, to confirm that the autofluorescence spill over does not create an artifact, we performed an additional test, the supravital 7-AAD analysis. We incubated cells with 7-AAD (1 h, room temperature) and recorded 7-AAD fluorescence and forward scatter (FSC). The long incubation permits a discrimination of viable cells which exclude the dye totally, from apoptotic cells that fluoresce dimly, and necrotic and late apoptotic cells that fluoresce brightly and are characterized by a decrease in FSC [28, 29].

To measure caspase activation, the Caspase-Glo® 3/7 luminescent assay (Promega) was used on cells incubated with different concentrations of M13_CC_-RB, 3 and 24 h post-irradiation. Control samples were kept in the dark. This single "add-mix-measure" reagent, was added to the photosensitized cell cultures resulting in cell lysis, followed by caspase cleavage of the substrate and generation of a “glow-type” luminescent signal recorded in an EnSpire (PerkinElmer) multimode plate reader, all according to the manufacturer instructions.

To monitor spheroids growth and homogeneity and to preliminary assess the eventual cytotoxic effect of M13_CC_-RB on multicellular tumor spheroids, CyQUANT® cell proliferation assay (Invitrogen, Thermo Fisher) was used, according to manufacturer’s instructions. Briefly, after treatment with M13_CC_-RB (45 min incubation) and irradiation (30 min) spheroids were incubated with the dye CyQUANT® GR properly diluted (400-fold) into the 1X cell-lysis buffer purchased in the kit and 20-fold diluted in distilled water prior to use. Solutions have been kept in the dark and used within a few hours of their preparation. Spheroids were then analyzed using the Nikon Eclipse Ti equipped with Digital Sight camera DS U3 (Nikon, Tokyo, Japan) or Nikon A1R confocal microscope (Nikon).

Spheroid viability was quantitatively assessed after treatment with phage M13_CC_-RB or RB alone at the same concentrations of RB equivalents (0.01 to 3 µM). Spheroids were incubated with phage M13_CC_-RB or RB for 45 min and irradiated for 30 min. After 24 h, the luminescent assay 3D CellTiter-Glo® (Promega, Madison, WI, USA) was employed to quantify the decrease in cell viability. Briefly, 100 µL of 3D CellTiter-Glo® were added to the complete medium 24 h after the treatment, solution was pipetted about 50 times for each well to disaggregate the spheroids. Luminescence was recorded using EnSpire multimode plate reader (PerkinElmer, Walthman, MA, USA). Data were normalized on the viability of untreated spheroids.

### Analysis of ROS generation in culture medium and intracellularly

To determine the M13_CC_–RB ability to produce peroxides upon visible light irradiation, Amplex® Red (AR) assay was used in a cell-free system. AR is a nonfluorescent and colorless molecule that reacts with peroxides, catalyzed by horseradish peroxidase (HRP), producing a fluorescent dye with an absorption maximum at 563 nm and an emission maximum at 587 nm, named resorufin. The number of generated peroxides was determined as the difference between resorufin produced by irradiated and not irradiated samples. The working solution (WS), containing AR and HRP, was prepared by mixing 1 mL of 50 mM PBS at pH 7.4 with 10 μL of AR 50 mM solution dissolved in DMSO and 10 μL of PBS solution of HRP 0.4 mg/mL. Sample solutions at different concentrations (0, 0.25, 0.5, 1, 2, or 4 μM) of RB and M13_CC_–RB were prepared in PBS 50 mM. Two identical 96 multiwell plates were prepared with the sample solutions (90 μL), using three technical replicates for each concentration. One plate was irradiated for 30 min with a white LED lamp (Valex cold white LED, irradiance on the plate surface = 24 mW/cm^2^, measured with the photo-radiometer Delta Ohm LP 471 RAD), while the second was kept in dark, as control. 10 μL of WS was then added to each well on both the plates. After the addition, the plates were incubated in the dark for 30 min, then the emission intensity was measured at 590 nm (λ_ex_ = 530 nm). Standard solutions of H_2_O_2_ were used to generate a calibration curve for converting the fluorescence intensity signal into the concentration of peroxides. Fluorescence measurements were carried out using a PerkinElmer EnSpire® Multimode Plate Reader.

To quantify the singlet oxygen (^1^O_2_) quantum yield (Φ_Δ_) of photoirradiated M13_CC_–RB, 9,10-anthracenediyl-bis(methylene) dimalonic acid (ABMDMA) was used as ^1^O_2_ detector in a cell-free system. The singlet oxygen produced in the solution upon visible light irradiation reacts with ABMDMA giving an endoperoxide and resulting in the bleaching of ABMDMA. The photoinduced generation of singlet oxygen was evaluated from the decrease in the absorbance at 400 nm. Briefly, 400 μL of iso-absorbing RB and M13_CC_–RB sample solutions were prepared in PBS, dissolved in D_2_O, at 15 μM of RB. 2 μL of a 5 mM ABMDMA stock solution in DMSO were added to each solution under investigation. The samples were then irradiated with a white LED lamp (irradiance on the cuvette surface = 2.4 mW/cm^2^) while maintained under stirring. Φ_Δ_ of M13_CC_–RB was determined using the following equation Φ_Δ_^S^ = k_S_/k_R_ × Φ_Δ_^R^ where k is the slope of the photodegradation rate of ABMDMA, S is the sample under investigation (M13_CC_–RB), R is the reference (RB), and Φ_Δ_^R^ is the ^1^O_2_ quantum yield of the reference (RB) which is known to be Φ_Δ_^R^ = 0.76 [30].

To assess the intracellular ROS production 20,000 HT29 cells were seeded in a 96 well-plate. Cells were treated and incubated in complete medium with increasing concentration of M13_CC_-RB (0.3–1 µM) at picomolar concentrations of bacteriophage or RB alone for 45 min. After incubation, cells were washed twice with PBS, to remove the excess of bioconjugated phage or PS. Cells were irradiated for 30 min in PBS. In parallel, to check eventual ROS generation in dark condition, cells were treated according to the before mentioned conditions and kept in the dark. After irradiation, 100 µL of ROSGlo (Promega) were added to each well and cells were incubated for 20 min at room temperature. Luminescence was read using EnSpire® Multimode plate reader (PerkinElmer).

### Microscopic analysis

The modified tropism of M13_CC_ toward CC cells was demonstrated by immunohistochemical confocal microscopy. 50,000 cells were seeded on round coverslips and grown overnight in the incubator. The cells were then incubated with or without 0.1 mg fibronectin (FN) (Superfibronectin, Sigma-Aldrich) for 30 min, followed by the addition of 10^12 phages (M13/M13_CC_) and incubation for 45 min. Unbound or excess phages were removed by washing twice with PBS, and the cells were fixed with 4% paraformaldehyde (PFA) in PBS for 15 min at room temperature. The cells were then washed with PBS + Tween 20 0.05% (Washing Buffer -WB) and permeabilized for 15 min with Triton 0.1%. Cells were incubated for 45 min with blocking solution (2% milk in PBS), washed with WB and incubated for 1 h in WB supplemented with mouse monoclonal anti-M13/fd/F1 filamentous phage (Progen) diluted 1:500. The cells were then washed three times with WB and incubated for 1 h in the presence of AlexaFluor568™ goat anti-mouse (Invitrogen). The excess of secondary antibody was removed by three washes with WB. Cells were stained for 15 min with Hoechst 33342 (1 µg/µL). Round coverslips were then washed and placed in an Attofluor cell chamber (Invitrogen) with 1 mL of PBS. Images were acquired with the Nikon A1R confocal microscope, and the laser settings were kept fixed for all images acquired.

Similarly, to assess specific retargeting of M13_CC_-RB towards CC cells, HT29 and DLD1 were seeded on round coverslips and grown overnight in the incubator. Next, cells were incubated with an equivalent phage concentration of 1 µM of RB in complete cell medium for 45 min at 37° C, washed and stained with Hoechst 33342 (1 µg/µL), used as nucleic dye, for 10 min. Round coverslips were then washed and fitted into an Attofluor cell chamber (Invitrogen) with 1 mL of 10% FBS and DMEM without phenol red supplemented with 1% penicillin/streptomycin. Images were acquired with Nikon A1R confocal microscope. Fluorescence quantification was performed on images acquired with Fiji free software.

Spheroid images were taken in bright field using Zeiss Axiovert 40 CFL microscope (Zeiss, Oberkochen, Germany) to evaluate the structure of each spheroid and select the most similar ones for further analyses. For the penetration evaluation, spheroids were incubated for 45 min in a single drop of complete McCoy cell medium added with M13_CC_-RB conjugate phage or RB alone at a concentration of 3 µM RB equivalents. Calcein and Hoechst 33342 were added to the spheroids at concentrations of 100 nM and 1 µg/µL, respectively.

Evaluation of penetration on fixed and optically cleared spheroids was performed as previously described by Nürnberg and colleagues with some modifications [31]. 3D cell cultures of HT29, DLD1 and HCT116 were generated, incubated with M13_CC_-RB for 45 min, washed thrice with PBS to remove unbound phages and then fixed with 4% PFA (Merck) for 1 h. Spheroids were then washed with PBS supplemented with FBS 1%, permeabilized with a solution of PBS and Triton 1% for 2 h with gentle shaking, and washed again with PBS supplemented with FBS 1%. Spheroids were then quenched for 1 h with 0.5 M glycine in PBS and then with a solution of 0.3 M glycine, 20% DMSO and 0.2% Tryton X-100 in PBS for 30 min. Samples were washed twice with PBS supplemented with FBS 1% and subjected to optical clearing with 88% glycerol for 18 h at room temperature. Spheroids were stained with 1 µg/µL of Hoechst 33342 and then observed with Nikon A1R confocal microscope using ND-acquisition z-stack.

For the conformational change evaluation to support the spheroid viability assay, samples were incubated with M13_CC_-RB conjugate phage or RB alone and Hoechst 33342 at the final concentration of 1 µg/mL. Samples were treated with low irradiance white light for 30 min as described before or kept in the dark. After 24 h, the spheroids’ structure was investigated in brightfield and with Nikon A1R confocal microscope using ND-acquisition z-stack to evaluate the penetration of the conjugated phage and the structural modifications.

### Flow cytometry

The tropism of M13_CC_-RB or free-RB to HT29 and DLD1 cell lines was evaluated by flow cytometry. Free-RB or bioconjugated phage were incubated in complete media, at the final RB concentration of 1 µM, with 500,000 adherent cells for 45 min. Unbound phages/sensitizer were removed by washing thrice with PBS and cells were detached using trypsin 1x. After trypsin inactivation with complete media, cells were washed with PBS, resuspended in 0.5 mL of PBS and analyzed with CytoFLEX S (Beckam Coulter). The fluorescence of at least 10,000 events was evaluated in the PE channel. Data analysis was achieved with CytExpert (Beckam Coulter) and FlowJo™ 10.0.7r2 version (Becton Dickinson, Franklin Lakes, NJ, USA).

### Statistical analyses

The results are expressed as mean ± SEM of at least three independent experiments. One or two-ways parametric Anova, or t-test were used for the comparison of the results and Dunnett or Tukei were used as post-tests. The statistical software GraphPad InStat 8.0 version (GraphPad Prism, San Diego, CA) was used and p < 0.05 was considered significant.

## Results

### Engineering of the M13_CC_ phage vector

To retarget the M13 phage vector to bind CC cells, we took advantage of a nine-amino-acid, disulfide-constrained peptide, CPIEDRPMC, isolated from a phage-display library on the HT29 CC cell line [23]. As such we surmised that the peptide could represent a valid moiety to redirect the tropism of the M13 phage to CC cells. The coding sequence of the peptide was cloned in frame with the pIII gene of a phagemid vector allowing for pentavalent display of the fusion on one phage end, using the Hyperphage (deltapIII) helper phagemid to supply for the remaining M13 genes (Fig. 1A). After phage production and purification (Materials and Methods), the M13_CC_ virions were validated for peptide display by Immunoblotting (Fig. 1A).

### Fibronectin antagonizes the specific binding of the engineered M13_CC_-vector to HT29 CC cells

Immunohistochemical confocal microscopy was used to assess the retargeting efficiency of engineered M13_CC_ towards HT29 and DLD1 cell lines (Fig. 1B–I and graph in Fig. 1J), as compared to controls without phage (Fig. 1B and C), and on two non-CC control lines, commonly used in cancer research, respectively isolated from the epidermis of a patient with epidermoid carcinoma (A431) or from a solid, invasive ductal carcinoma from a breast cancer patient (BT474)(Supplementary Fig S1A–S1C). Results showed that M13_CC_ effectively targeted both CC cell lines (Fig. 1F and G), with a stronger preference for HT29, while the binding to A431 and BT474 resulted significantly weaker (Supplementary Fig. S1D). Experiments using an unmodified wild-type M13 phage showed no differences in comparison to the control (Fig. 1D and E), demonstrating that the specific binding of M13_CC_ to CC cells is mediated by display of the CPIEDRPMC peptide. Preincubation with FN was previously shown to inhibit binding of the targeting peptide to CC cells [24]. As such we investigated in parallel the antagonistic effect of FN on the M13_CC_ tropism (Fig. 1H, I). Pre-treatment of both cell lines with FN impaired the binding of M13_CC_ especially on the HT29 CC cell line (Fig. 1J), in accordance with previous observations.

### The M13_CC_-RB bioconjugate vector targets preferentially HT29 CC cells

**Fig. 2:**
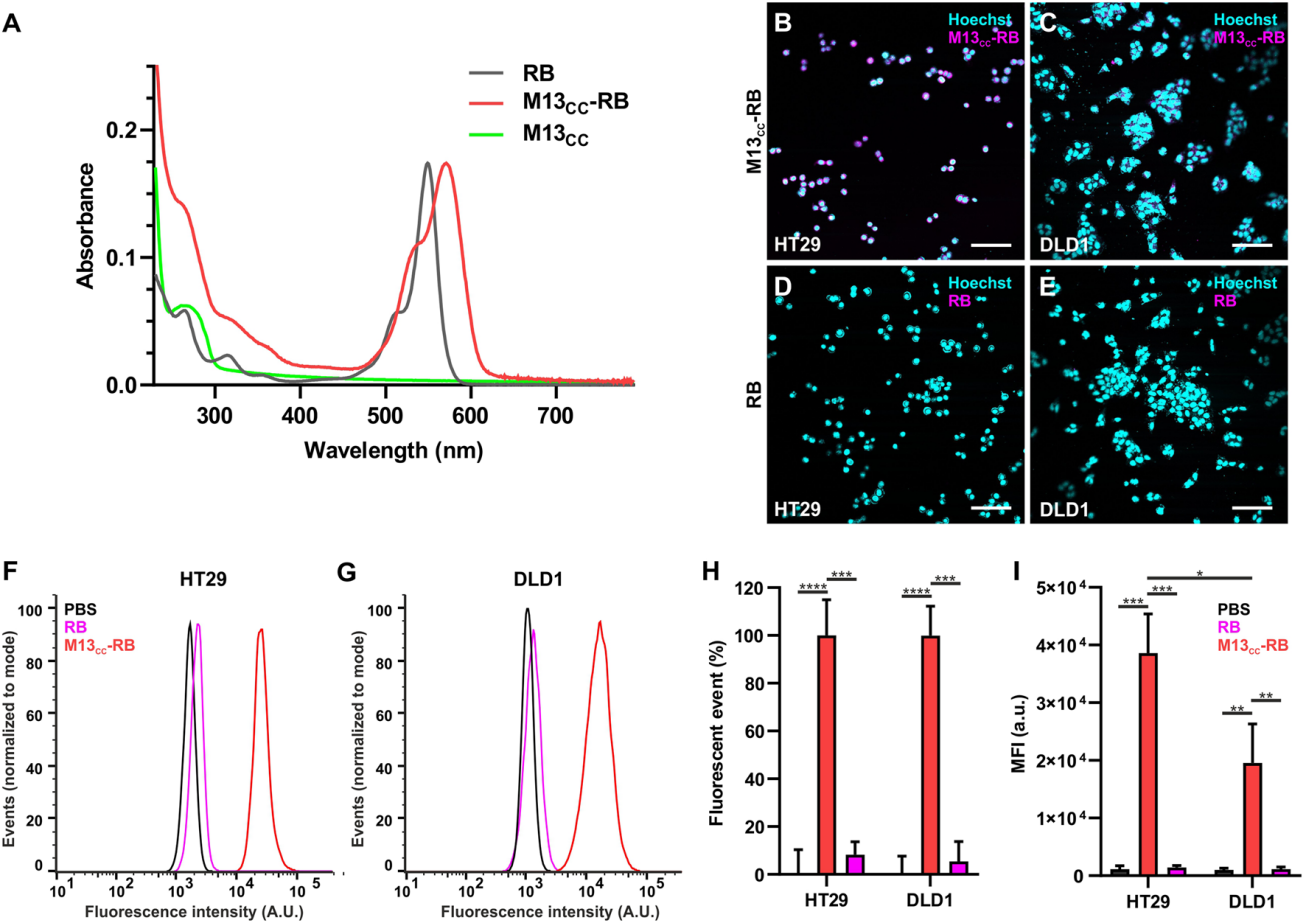
Enhanced targeting of the M13_CC_-RB towards CC cells, significantly higher for HT29 cells. **A** UV–VIS absorption spectra of RB (grey), M13_CC_-RB (red) and M13_CC_ (green). (B) Microscopic analysis of the retargeting properties of the engineered phage 1 µM RB equivalents tested on **B** HT29 and **C** DLD1 CC cell lines. Microscopic analyses were performed also with free RB on **D** HT29 and **E** DLD1. Flow cytometry analysis of the retargeting ability of either free RB (pink) or M13_CC_ -RB (red) on **F** HT29 and **G** DLD1, PBS was used as control (in black). **H** Percentage of fluorescent events calculated via flow cytometry. **I** Mean fluorescence intensity of HT29 cells and DLD1 cells incubated with free RB (in pink), M13_CC_-RB (in red) and PBS (in black). All graphs and calculations were performed using FlowJo™. Statistical significance was calculated by one-way parametric ANOVA followed by Dunnet’s multiple comparison test * p < 0.05; *** p < 0.001; **** p < 0.0001; n = 3

Next, RB was covalently bioconjugated to the M13_CC_ virions using the EDC/NHS cross coupling reaction, in which the activated carboxylic-acid group of RB reacts with the accessible N-terminal and lysine lateral group of M13_CC_ capsomers, producing a covalent amidic bond. UV–Vis characterization (Fig. 2A) of the bioconjugate showed the characteristic bathochromic shift and a broadening of the RB absorption spectra, compared to RB alone. These spectroscopic changes confirmed the bioconjugation of the RB PS to the M13_CC_ capsid. Based on the extinction coefficient of RB, we estimated an average number of 910 RB molecules per phage virion.

The tropism of M13_CC_-RB was investigated on the two colon cancer cell lines, HT29 and DLD1. The retargeting ability of the RB phage bioconjugate vector was further validated by confocal microscopy and flow cytofluorimetry taking advantage of the intrinsic fluorescence of the RB sensitizer. Confocal microscopy experiments and semi-quantitative analyses showed that the retargeted M13_CC_-RB phage bioconjugate binds with higher affinity to the HT29 line (Fig. 2B) as compared to DLD1 (Fig. 2C), while bare, unconjugated RB did not bind the cells per se (Fig. 2D and E, respectively for HT29 and DLD1). The results demonstrated that the retargeted phage-RB bioconjugate is able to vector the sensitizer towards both cell lines, but significantly better towards HT29 cells, in accordance with the results obtained for the unconjugated M13_CC_. Additionally, flow cytometry analyses were conducted on both cell lines to confirm the retargeting ability of the engineered and bio-conjugated phage. The shift of the peak of the fluorescence intensity (Fig. 2F and G) compared to the controls (incubated only with PBS or RB) demonstrated the retargeting ability of the vector on both cell lines. Furthermore, the fluorescent population of both CC cell lines was found to be 100% (Fig. 2H), indicating successful targeting. The mean fluorescence intensity (MFI) was significantly higher for HT29 cells than for DLD1 cells (Fig. 2I), in line with the observations from the confocal microscopy experiments. These results demonstrate that conjugation with the RB sensitizer did not significantly impair the tropism of the phage vector, maintaining a better tropism towards HT29 cells.

### Selective photodynamic killing of CC cells is mediated by M13_CC_-RB upon light irradiation

**Fig. 3:**
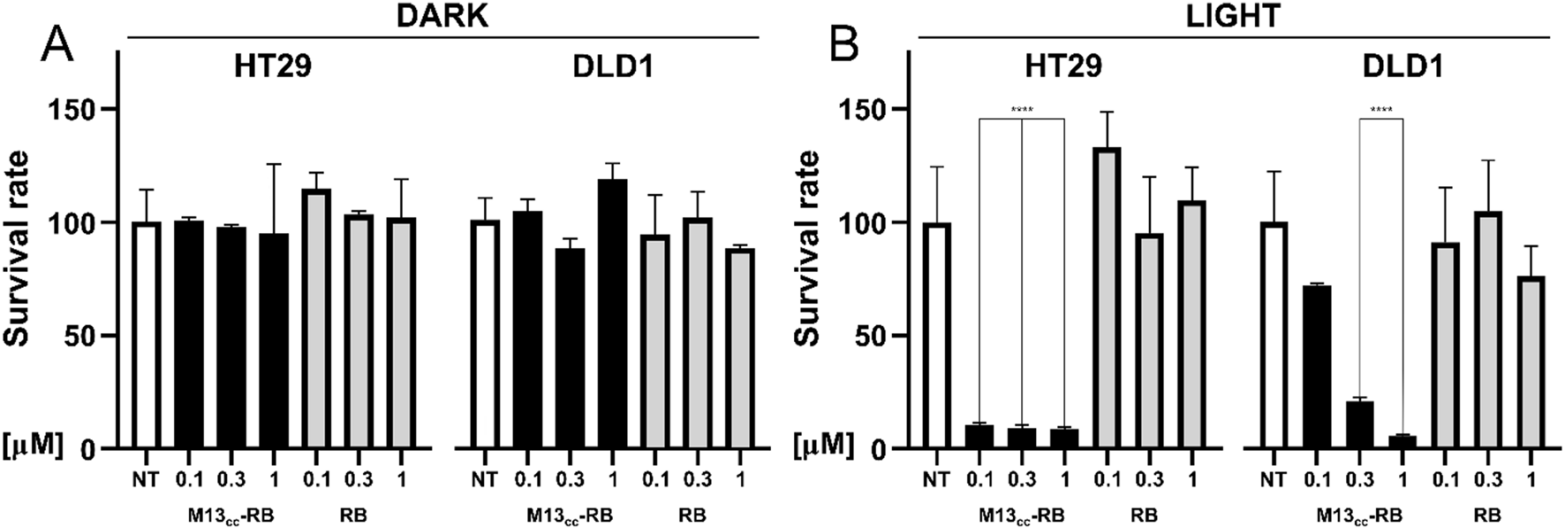
Photoirradiated M13_CC_-RB strongly decreases CC cell viability. Cytotoxic effects of M13_CC_-RB or RB alone on HT29 and DLD1 cells kept in the dark (DARK, **A**) or irradiated for 30 min with a white led bulb (LIGHT, **B**), 24 h after treatment. The same sensitizer concentrations (RB equivalents) were used to compare M13_CC_-RB and RB alone. Cell viability was analyzed using MTT test on three independent biological replicates (n = 3). Statistical significance was calculated by one-way parametric ANOVA followed by Dunnet’s multiple comparison test; * p < 0.05; *** p < 0.001; **** p < 0.0001 compared to untreated cells (NT)

Next, to test the photodynamic activity and specificity of the phage nanovector, the high-targeted HT29 and low-targeted DLD1 CC cells were incubated with increasing concentration of M13_CC_-RB or RB alone. To compare results, the same sensitizer concentrations between the M13_CC_-RB bioconjugate and RB alone were used (same RB equivalents). After washes, cells were irradiated with a low power led bulb for 30 min; controls were kept in the dark. While dark toxicity was completely negligible and RB alone showed an extremely low cytotoxic activity (Fig. 3A), a significant concentration-dependent decrease in cell viability was observed after treatment with photoirradiated M13_CC_-RB on both HT29 and DLD1 cell lines (Fig. 3B). Moreover, the retargeted vectors almost completely killed HT29 cells already at the lowest concentration tested (0.1 µM RB equivalents, 8% ± 0.8 viable cells), whereas the observed cytotoxic activity was much lower on the DLD1 cells at the same concentration (72% ± 0.9 viable cells). Notably, this effect cannot be attributed to a different photo-sensitivity of the tested cell lines, as both HT29 and DLD1 exhibit similar viabilities upon irradiation without sensitizer or phage. Thus, although the decrease in cell viability was comparable at higher concentrations of sensitizer equivalents (0.3 and 1 µM RB equivalents, Fig. 3B), HT29 cells resulted more sensitive to phage-mediated PDT treatment, in line with the better targeting of M13_CC_-RB to this cell line (Fig. 2E).

While the CPIEDRPMC peptide was shown to bind with higher affinity to both HT29 and DLD1 cell lines, it also displayed poor binding affinity to the HCT116 CC cell line [23]. Accordingly, additional photodynamic experiments were carried out to test the efficacy of the M13_CC_-RB bioconjugate against this cell line. Results showed that HCT116 cells are killed less efficiently than the HT29 cell line after irradiation, with survival rates comparable to the DLD1 cell line (Supplementary Figure S2), which is targeted worse than the HT29 cells (Figs. 1J and 2I). This is consistent with the low affinity binding of the CPIEDRPMC targeting peptide to the HCT116 cell line and correlates the elicited photosensitization with the targeting efficiency of the phage vector.

Together the results demonstrate that i) the engineered phage nanovector can efficiently vehiculate light-triggerable PS molecules to CC cells and promote photodynamic cytotoxic effects already at picomolar concentrations of the vector; ii) the cytotoxic effects are more pronounced on the HT29 cell line that is targeted with better efficiency, suggesting a photosensitization specificity provided by the phage vector.

### Intracellular ROS are generated upon M13_CC_-RB irradiation

**Fig. 4:**
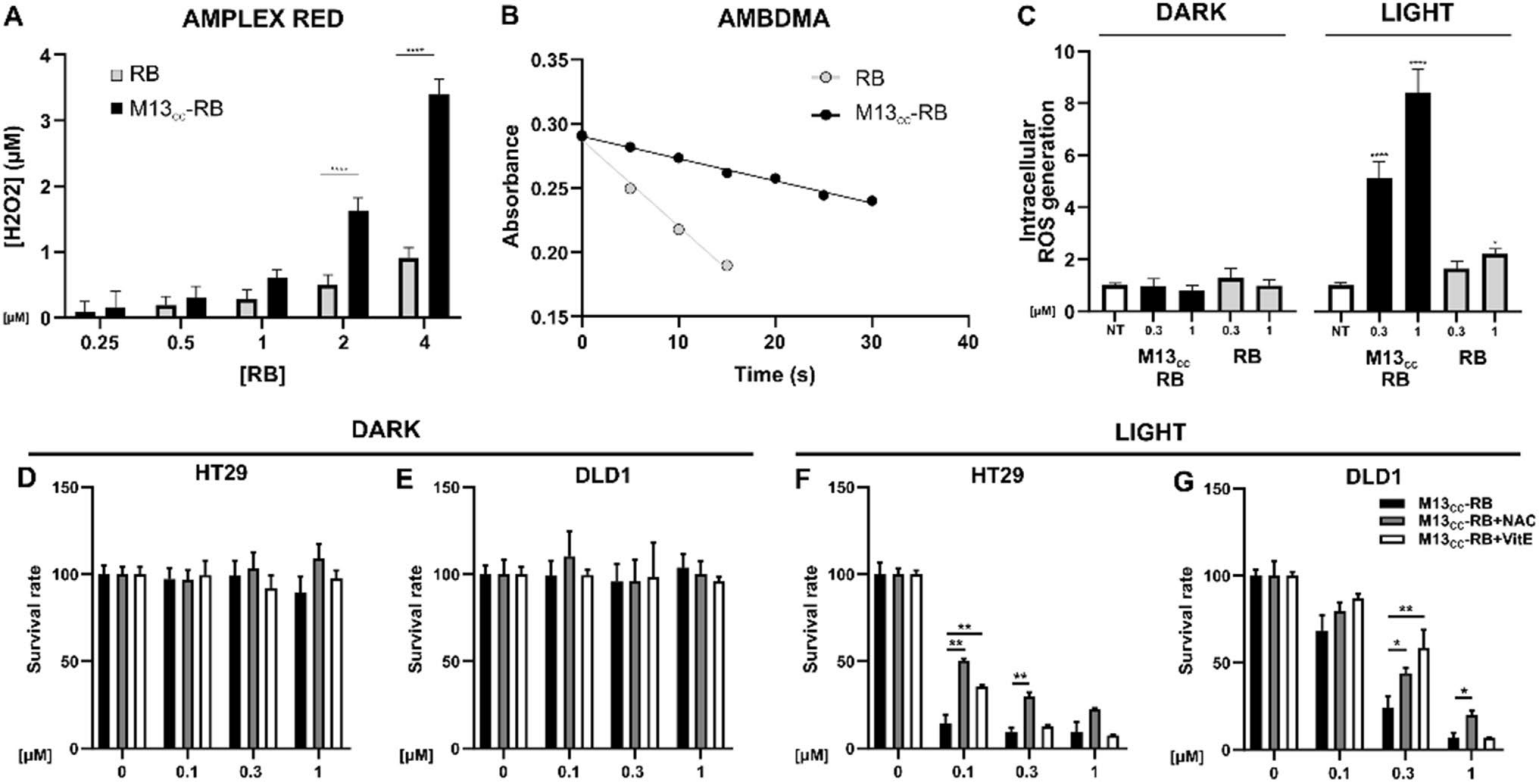
M13_CC_-RB increases ROS intracellular levels after irradiation. **A** Quantification of peroxides, assayed by AR test, produced by different concentrations of RB (white squares) and M13_CC_-RB (black squares) after 30 min of white LED irradiation. **B** Singlet oxygen generation produced by RB (grey dots) and M13_CC_-RB (black dots) at increasing irradiation times, monitored by the decrease of UV–Vis absorption of ABMDMA. **C** Intracellular ROS generation measured with ROS-Glo assay (Promega) after exposure to M13_CC_-RB in presence or absence (dark) of irradiation (30 min) of HT29 cells on three independent biological replicates (n = 3). Statistical significance was calculated by two-way ANOVA followed by Dunnet’s multiple comparisons test. * p < 0.05; **** p < 0.0001 compared to untreated cells (NT). PDT with M13_CC_-RB in presence of ROS inhibitor was evaluated on **D,F** HT29 and **E,G** DLD1 cells. Cells were treated with M13_CC_-RB in presence of PBS (black bars), NAC (grey bars) or vitamin E (VitE, white bars) and then **D,E** kept in dark conditions or **F,G** irradiated. Statistical significance was calculated by one-way parametric ANOVA followed by Dunnet’s multiple comparisons test; * p < 0.05; ** p < 0.01; (n = 3)

To elucidate the mechanisms underpinning the light-dependent cytotoxicity of M13_CC_-RB, the ROS generation mediated by the phage vector was first investigated in a cell-free system. The photodynamic performances of M13_CC_-RB were evaluated using respectively the Amplex Red (AR) assay to detect peroxides (Fig. 4A) and ABMDMA to detect ^1^O_2_ production (Fig. 4B). The AR assay performed to isoabsorbing solutions of RB, with concentrations ranging from 0.25 to 4 µM, suggested that the bioconjugation to the bacteriophage significantly enhanced peroxides production by RB, as compared to the free PS. Conversely, the singlet oxygen production exhibited by M13_CC_-RB (Φ_Δ_ = 0.20) resulted less efficient with respect to an equimolar solution of free RB (Φ_Δ_ = 0.76). As a control, the same experiments were replicated in dark conditions, where both the assays did not exhibit ROS generation activity, confirming the light-dependency for their generation (data not shown). These results indicate an improved ability to generate peroxides of the M13_CC_-RB vector, in line with previous observations made on a similarly RB-conjugated M13 scaffold retargeted to the EGFR receptor [22].

To demonstrate the capacity of the phage vector to mediate intracellular ROS accumulation, important for PDT efficiency, a sensitive bioluminescent assay that measures the level of hydrogen peroxide directly in cell culture was used (ROS-Glo™). HT29 cells were incubated with 0.3 and 1 µM M13_CC_-RB, irradiated, and generated ROS levels were compared to free RB. A sevenfold-increase in intracellular ROS levels was observed at the highest phage concentration tested, while the increase in intracellular ROS mediated by free RB resulted negligible at the same conditions. Moreover, ROS generation in the dark was not observable under all conditions tested (Fig. 4C). To validate the involvement of ROS generation in triggering the death of CC cells, photodynamic experiments in media supplemented with ROS inhibitors (antioxidants) were carried out. In particular, HT29 and DLD1 cells were grown and treated in media containing respectively 10 mM NAC, a common antioxidant which protects cells against a wide range of pro-oxidative insults, or 100 µM vitamin E, a fat-soluble antioxidant which protects cell membranes from ROS. Results showed similar survival rates in dark conditions for both HT29 and DLD1 cell lines (respectively Fig. 4D and E), while a significant effect of both NAC and vitamin E was observed in protecting both CC cell lines from lethal photosensitization (Fig. 4F and G). These results strongly suggest that the cytotoxic effects mediated by M13_CC_ phage vector are also attributable to intracellular accumulation of ROS, promoting cell death.

### M13_CC_-RB irradiation triggers necrosis

**Fig. 5:**
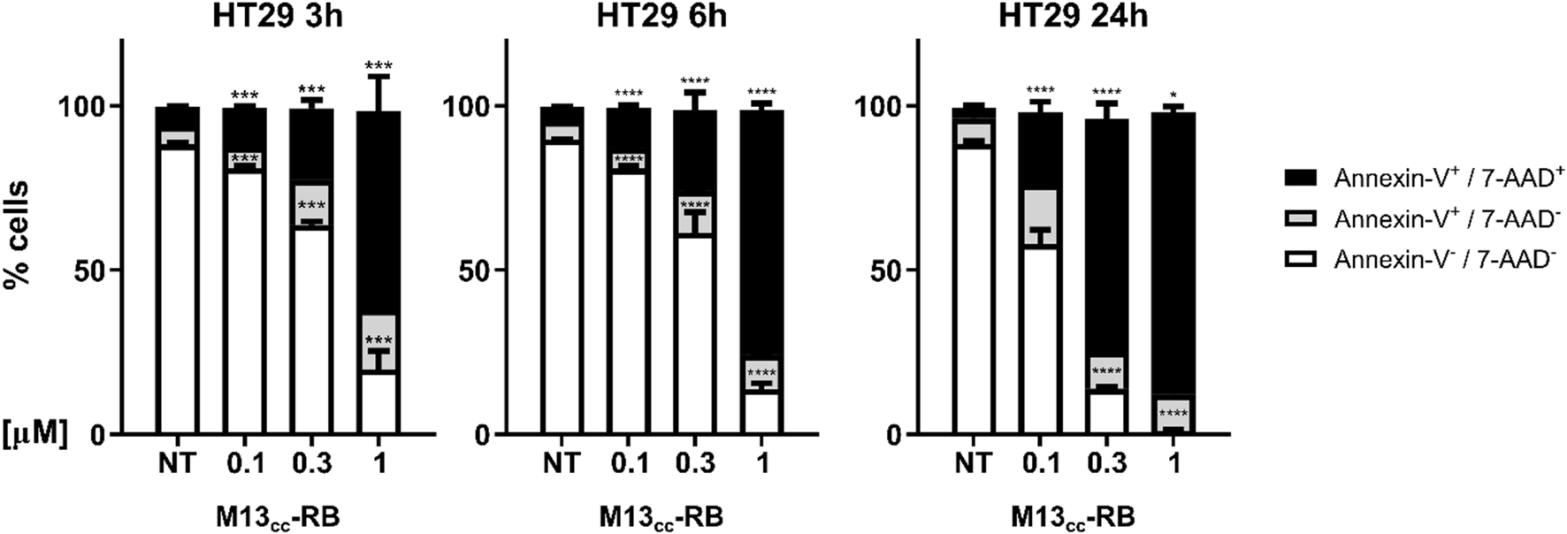
Cell death mechanisms induced by M13_CC_-RB mediated photosensitization. Percentage of Annexin-V^−^/7-AAD^−^, Annexin-V^+^/7-AAD^−^, Annexin-V^+^/7-AAD^+^ HT29 cells after 3, 6 or 24 h from treatment with photoactivated M13_CC_-RB on three independent biological replicates (n = 3). Statistical significance was calculated by two-way Anova followed by Dunnet’s multiple comparisons test. * p < 0.05; *** p < 0.001; **** p < 0.0001 compared to untreated cells (NT)

Cellular exposure to specific oxidative stressors can trigger distinct molecular cell death mechanisms (e.g. necrosis, apoptosis, autophagy, etc.) [32, 33]. The non-permeant dye 7-AAD was used as marker of membrane integrity, allowing to distinguish between cells in the early regulated cell death stage (Annexin-V^+^/7-AAD^−^) or cells with damaged cell membrane (Annexin-V^+^/7-AAD^+^). Regardless of post-irradiation times, most dying HT29 cells resulted Annexin-V^+^/7-AAD^+^ in a dose-dependent manner, indicating necrosis (Fig. 5). Indeed, the percentage of double positive cells significantly increased starting from the lowest RB equivalents concentration (0.1 µM) and after the shortest post-irradiation time tested (3 h, 11.7% ± 0.8). A further gain in cytotoxicity and necrotic events was observed with increasing M13_CC_-RB vector concentrations or elongating the time of sampling after irradiation. At maximum RB equivalents and up to 24 h post-irradiation, the majority of HT29 cells (85.8% ± 3) presented evidence of a damaged cell membrane (Fig. 5). We recorded the same mechanism of cell death also at shorter irradiation time (10 min), demonstrating that necrosis still prevails over apoptotic events (Supplementary Figure S3) and that irradiation time does not drive the choice of the cell death mechanism triggered. The necrotic nature of cell death induced by M13_CC_-RB was further confirmed by the morphological analysis of CC cells via flow cytometry, which indicate a high percentage of necrotic events as shown in Supplementary Figure S5. To further support the low levels of apoptotic cell death, a luminescent caspase assay was performed, 3 and 24 h after incubation with M13_CC_-RB phages and irradiation (Supplementary Figure S4). At 3 h post-irradiation and lower RB equivalents caspase 3/7 activity increased marginally (< 1.3 × fold). On the contrary, at 1 μM RB equivalents, corresponding to the highest level of light-dependent cytotoxicity elicited, a marked decrease in caspase activity was recorded (Supplementary Figure S4A). This can be explained by the higher number of cells killed by an apoptotic-independent mechanism following photodynamic triggering of the RB sensitizer. At 24 h post-irradiation an even stronger decrease in caspase 3/7 activity was observed, in line with reduced number of cells persisting after lethal photosensitization (Supplementary Figure S4B). These results indicate that M13_CC_-RB triggers principally necrotic cell death following irradiation.

### M13_CC_-RB deeply penetrates multicellular CC spheroids

Experimental 3D models aim to bridge the gap between conventional cell cultures and in vivo experiments, recapitulating the architectural, biochemical and biomechanical cues, as well as cell–cell interactions more faithfully than 2D cultures [34]. They also proved valuable to provide novel insights into the therapeutic mechanisms of PDT [12, 35]. The penetration of the retargeted phages in a CC 3D model was the first step verified before testing the efficacy in PDT, since poor diffusion within the inner core of the tumor influences the response to anticancer treatment. To test the activity of the phage nanovector platform on 3D models, we first validated the reproducibility of CC spheroid generation in terms of dimension and shape from bright field microscopy acquisitions and 3D-reconstructions (data not shown). The spheroids were further inspected after 5 days of growth with CyQUANT® fluorescent dye to visualize actively proliferating cells: only the outer layer of the CC spheroids stained positive for the dye (data not shown), indicating a deeper non-proliferating core surrounded by a replicating cell layer.

**Fig. 6:**
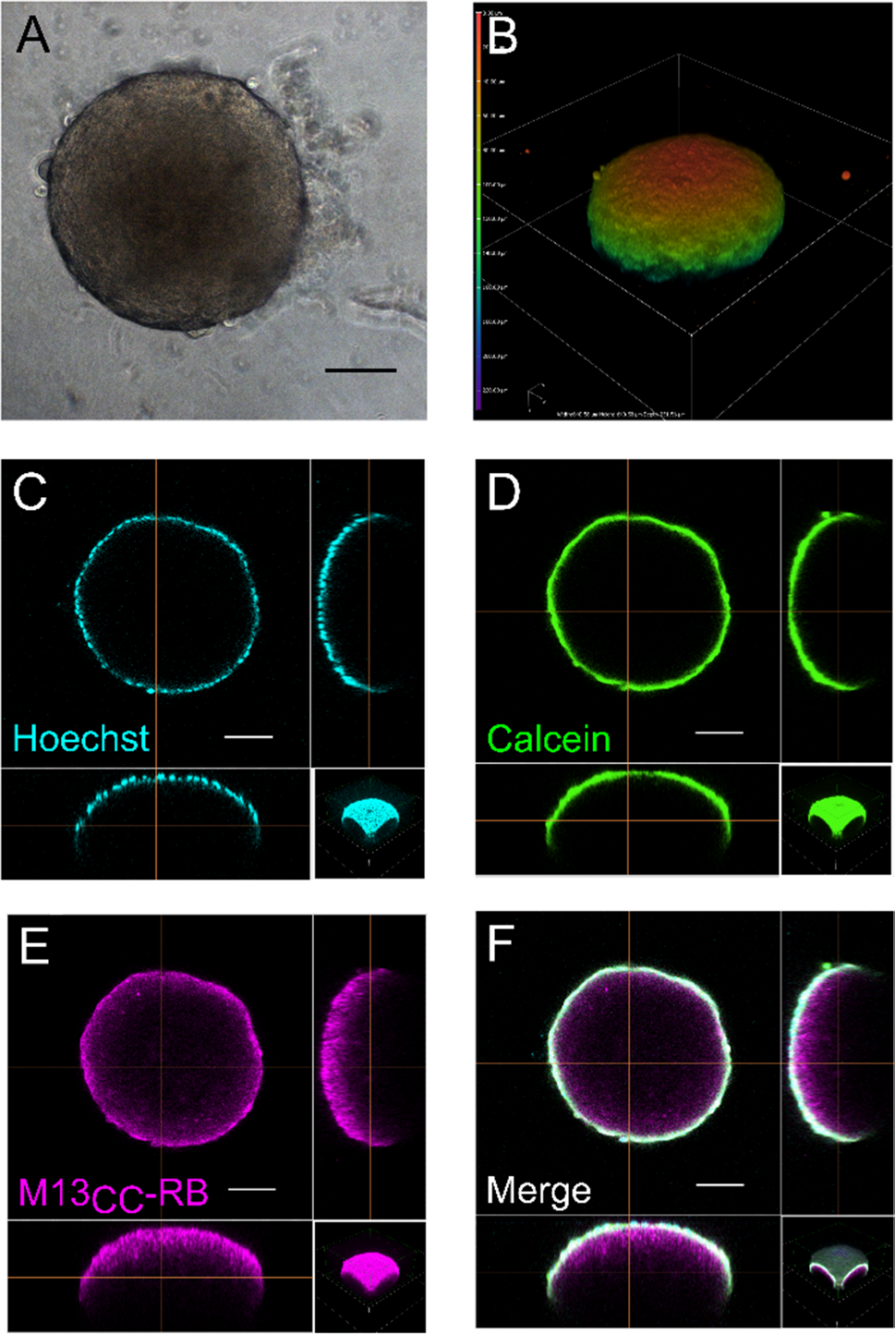
M13_CC_-RB deeply penetrates CC multicellular spheroids. Evaluation of multicellular spheroid conformation in **A** bright field and **B** confocal microscopy after 24 h from incubation with M13_CC_-RB at 3 µM concentration of PS. The heatmap highlights the Z-depth coding, showing the relative height of cells in the confocal stack, demonstrating the integrity of the CC spheroid. Spheroid penetration of M13_CC_-RB: **C** Hoechst, cyan; **D** Calcein AM, green; **E** M13_CC_-RB, magenta; **F** merge. Scale bars = 100 µm

To investigate the interaction of the phage vector platform with CC spheroids, M13_CC_-RB phages were incubated with HT29 spheroids for 24 h in absence of photoactivation. Comparison of bright field images taken before and after (incubation showed that the vector per se had no impact on the spheroid shape and integrity (Fig. 6A and B). The CC spheroids were then systematically inspected at the confocal microscope, taking advantage of the intrinsic fluorescence of RB to localize the phage vector, together with Hoechst (Fig. 6C) and Calcein AM (Fig. 6D) live stains to detect actively proliferating cells within the spheroid. Strikingly, a deep penetration of the M13_CC_-RB nanovector could be observed also into the non-proliferating spheroid core (Fig. 6E), otherwise inaccessible even to permeant low molecular nuclear stains such as Hoechst (Fig. 6F). Again, no conformational changes were evidenced in the 3D reconstruction of the phage-penetrated spheroid (Fig. 6B). These results demonstrate the unforeseen capacity of the M13_CC_-RB vector to thoroughly penetrate the CC spheroid, carrying the PS load deep-down into the spheroid core, where other therapeutic molecules or platforms (e.g., antibodies, virus-like particles etc.) generally fail to deliver.

These results were further supported by experiments on transparized spheroids on three different CC cell lines: HT29, DLD1 and HCT116 (Supplementary Figure S6). A deeper staining of Hoechst could be observed for all the spheroids; yet an even deeper penetration of the M13_CC_-RB phage could be detected, especially for the HT29 line, which is targeted with higher efficiency by the retargeted phage (Figs. 2, 3, 4 and Supplementary Fig. S2). These results confirm the superior spheroid penetrating features of the phage vector and also indicate how the targeting specificity is maintained between 2 and 3D models.

### M13_CC_-RB disrupts CC spheroids, impairing their viability

**Fig. 7:**
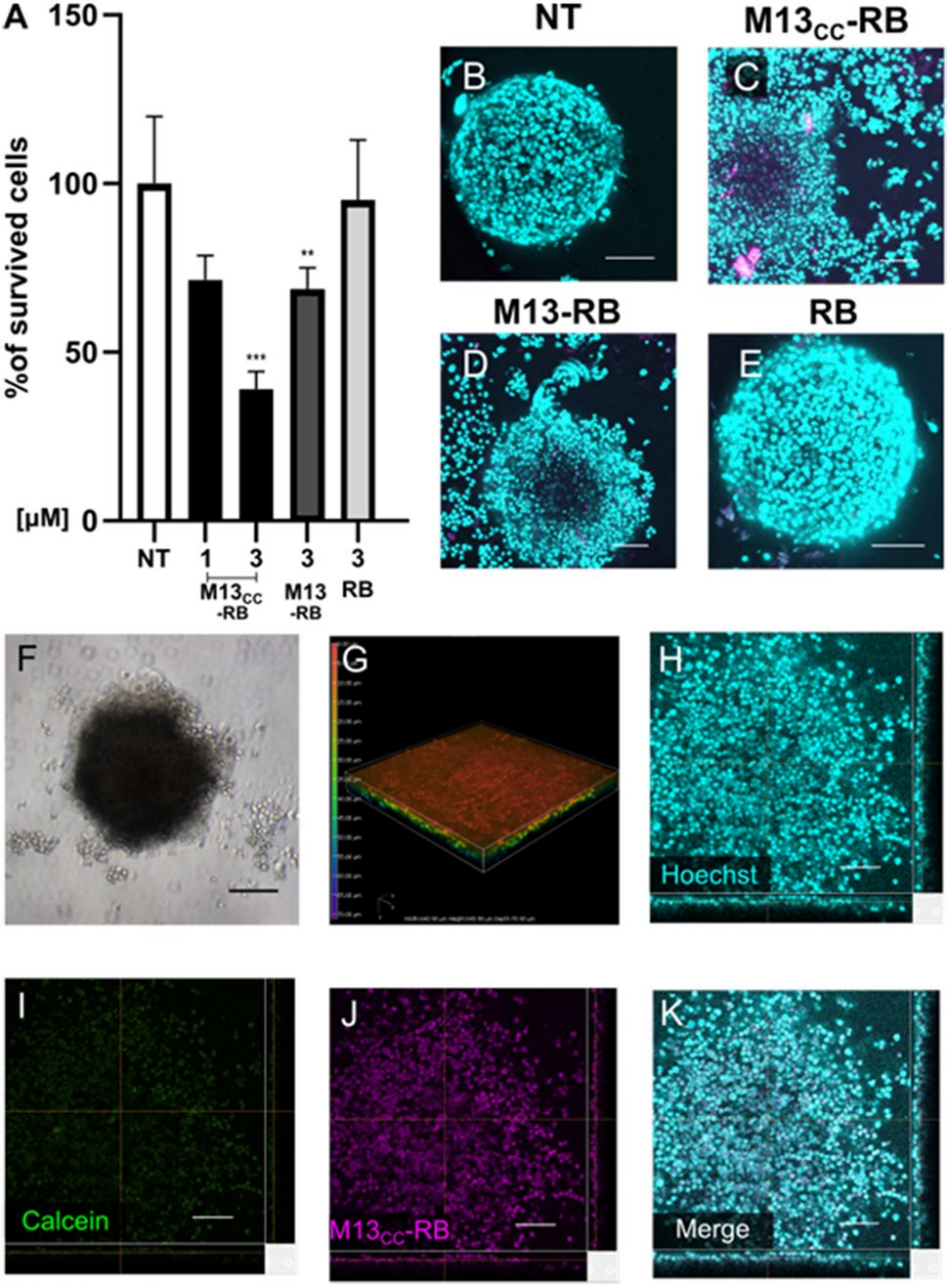
M13_CC_-RB impairs CC multicellular spheroid structure and viability upon irradiation. **A** Cytotoxic effects of M13_CC_-RB (1 µM and 3 µM), M13-RB (3 µM) or RB alone (3 µM) after 30 min of irradiation. 3D CellTiter-Glo® was used as viability assay on three independent biological replicates (n = 3). Statistical significance was calculated by one-way ANOVA followed by Dunnet’s multiple comparison test; ** p < 0.01; *** p < 0.001 compared to untreated cells (NT). **B** Untreated control spheroid; **C** spheroid disaggregation after M13_CC_-RB-mediated PDT; **D** partial spheroid disaggregation after PDT with M13-RB; **E** lack of spheroid disaggregation after PDT with RB alone. Evaluation of multicellular spheroid conformation in **F** bright field and **G** confocal microscopy after PDT with M13_CC_-RB at 3 µM concentration of PS. The heatmap highlights the Z-depth coding, showing the relative height of cells in the confocal stack, and demonstrating loss of spheroid integrity. Spheroid disaggregation and loss of viability after M13_CC_-RB photoactivation: **H** Hoechst, cyan; **I** Calcein AM, green; **J** M13_CC_-RB, magenta; **K** merge. Scale bars = 100 µm

To test the cytotoxic effects of the photoactivated M13_CC_-RB on the 3D model, as compared to RB alone, multicellular CC spheroids were incubated with the RB-conjugated phage or free PS (1 to 3 µM RB equivalents), irradiated for 30 min and tested for spheroid viability with the 3D CellTiter-Glo® reagent. A significant decrease in cell viability was recorded for the M13_CC_-RB 3 µM (39% ± 5 viable cells), while weaker cytotoxic effects were recorded with lower RB equivalents (M13 _CC_-RB 1 µM; 71% ± 7 viable cells) or with a wild-type (non-retargeted) M13-RB phage (Fig. 7A). No photosensitization was observed for RB alone under the same conditions (Fig. 7A). These results suggested that RB alone and the wild-type M13 phage are not capable of fully permeating the multicellular CC spheroid. On the contrary, recalling also the limited radius of action of ROS from the site of generation (5–20 nm), the engineered phage is capable of vectoring high payloads of the RB PS within the spheroid, promoting significant CC cell death upon irradiation. To further support the potential of M13_CC_-RB-mediated photosensitization, the conformation of irradiated spheroids was inspected by confocal microscopy (Fig. 7B–E). M13_CC_-RB treated spheroids appeared flattened with a complete loss of three-dimensional structure (Fig. 7C). The collapse of the spheroid was less evident after treatment with the M13-RB phage bioconjugate (Fig. 7D), whereas control spheroids (Fig. 7B) or spheroids exposed to RB alone (Fig. 7E) appeared intact. The complete loss of 3D structure of the spheroid after M13_CC_-RB incubation and photoirradiation was further validated in bright field microscopic analysis of representative samples 24 h after irradiation (Fig. 7F). The loss of spheroid shape and architecture was further supported by the 3D reconstruction of the confocal z-stack of M13_CC_-RB treated spheroids 24 h post-irradiation (Fig. 7G). The same spheroids were then inspected, for M13_CC_-RB, Calcein AM and nuclear staining (Hoechst) (Fig. 7H–K). Sheer disruption of the spheroid architecture was observed, with hundreds of disaggregated cells staining for Hoechst (Fig. 7H), with scattered RB fluorescence highlighting the presence of the residual phage structures (Fig. 7J). Interestingly the disaggregated cells failed to display Calcein AM fluorescence staining (Fig. 7I). This result strongly indicates that the cells of the disrupted CC spheroid lost viability.

## Discussion

CC overwhelming morbidity and mortality has a huge impact on human health and consequently on healthcare systems, calling for innovative therapeutic and preventive strategies to reduce its incidence. PDT has not been well characterized for the management of CC, despite the promising results in early clinical case series [6]. New approaches to foster the potential of PDT in CC tumor models will help to boost PDT applications as adjuvant CC therapy.

Our results report promising translational features of an engineered phage nanovector targeting CC cell lines. The phage vector was retargeted through phage display of a peptide nonamer binding to CC cells [23, 24] leading to lethal photosensitization of CC cells, in a dose and target-specific manner. The efficacy of this approach was demonstrated also on a multicellular 3D model of CC spheroids, showing vector penetration and lethal photosensitization of CC spheroids. In the absence of light irradiation, no cytotoxicity was observed, confirming the excellent tolerability of the phage vector PS bioconjugate.

M13 represents a promising platform to combine phage display technology with photochemistry, due to the peculiar characteristics of this filamentous phage. Indeed, when compared to other PS targeting agents, the use of M13 phage offers several advantages, such as enhanced binding affinity due to pentavalent display of the targeting moiety on the minor coat protein pIII, cost-effective production, and higher PS payload as a result of multiple functionalization sites on the major capsid protein pVIII [20, 36]. Notably the M13 platform could in principle be engineered to display other targeting moieties reported to specifically bind to CC cells [8, 37, 38], or to vehiculate different sensitizers excitable by the deeper penetrating near-infrared (NIR) or ultrasound radiation. Thus, the platform provides a wide flexibility for therapeutic approaches, beyond the choice of modifications presented in this work. Up to date, several PS were approved for clinical applications or are under clinical trials for the treatment of the precancerous lesion of actinic keratosis, advanced cutaneous T-cell lymphoma, basal and squamous cell skin cancer esophageal cancer and non-small cell lung cancer [14]. After the first approved Photofrin® or Temoporfin, belonging to the first and second generation, respectively, a third generation of PS is emerging, with the aim to improve cancer-targeting efficiency by chemical modification, nano-delivery systems, or antibody conjugation [39].

Due to the high performance of RB as photosensitizer, we selected this PS for the bioconjugation with M13_CC_. RB is a fluoresceine derivative, containing multiple halogens (i.e., chlorine, iodine) with high atomic number substituents. Due to the heavy atom effect, the intersystem crossing rate is significantly enhanced, generating the RB triplet excited state that is crucial for ROS generation [30]. In addition, RB is cheap, widely available, and biocompatible. Besides, RB has been designed as an orphan drug by FDA for the treatment of metastatic melanoma, hepatocellular carcinoma, neuroblastoma, and ocular melanoma. However, RB therapeutic potential is poorly exploited due to its hydrophilic tendency, caused by the two negative charges at physiological pH, and its reduced half-life, and both these characteristics have an unfavorable impact on its uptake and biodistribution [25]. Engineered M13 therefore represents the ideal nanovector to vehicle the RB inside target cells, as it does not appear to affect the photochemical performances of the PS, while being able to carry effective PS payloads to cancer cells already at picomolar concentrations of the vector. This is anticipated to increase the efficiency of PDT, not only in terms of tumor ablation, but also to prompt photoimmunogenicity, a light-dependent immunogenic form of cell death, able to generate a strong and long-lasting anti-cancer immunity [11, 40]. In this context, RB acetate PDT already showed its ability to induce exposure and/or release of damage associated molecular patterns (DAMPs), responsible for immunogenic cell death [41], making this PS a good candidate to prompt immunogenic anticancer response [42].

After conjugation, the M13_CC_-RB bioconjugate was tested for its in vitro PDT efficacy on different CC cell lines, (HT29, DLD1 and HCT116). HT29 cells showed higher sensitivity to PDT treatment compared to DLD1 at the lowest tested concentration of PS, corresponding to picomolar concentrations of M13. However, at highest concentrations of treatment, photoirradiated M13_CC_-RB almost completely impaired cell viability in CC tested cell lines. These CC cells differ in the expression of the DNA repair system MMR, proficient for HT29 and deficient for DLD1 and for HCT116. MMR is responsible for DNA proofreading, repairing, and removing DNA base-errors during its replication [43]. It is well recognized that MMR-deficient CC cells are more resistant than MMR-proficient cells to anticancer therapy, such as 5-fluorouracil [44], supporting a higher responsiveness of HT29 also to other anticancer treatments. Moreover, the better efficacy of irradiated M13_CC_-RB in HT29 cells is supported by the better retargeting of the bacteriophage to this cell line compared to DLD1, as verified by both microscopic and flow cytometric analyses (Figs. 1 and 2). However, also DLD1 and HCT116 cells undergo significant cell death by the photoactivated functionalized bacteriophage at the highest tested concentrations, acknowledging PDT as promising strategy also for MMR-deficient tumors [45].

Preincubation with FN showed an antagonistic effect on the binding of the phage to CC cells, particularly against the HT29 line (Fig. 1). This result tentatively suggests the involvement of the FN receptor (FNR) in the specific binding of the M13_CC_ phages displaying the CPDIERPMC moiety, in line with previous published results for the same peptide [24]. In prospect, the FNR receptor (or integrin α5β1) represents an interesting target for PDT, because alterations of the α5β1 expression pattern have been related to progression, proliferation, angiogenesis, and metastatisation in a considerable number of human carcinomas, including CC, where it associates with poor prognosis for patients [46]. Accordingly, the targeting of FNR with specifically designed nanovectors could significantly help in inhibiting tumor growth and tumor metastasis, decreasing side effects associated with different cancer therapy [47].

Several approaches have been taken to use engineered bacterial viruses as cancer therapeutics [21, 22, 48], with remarkably interesting declinations involving the use of transmorphic hybrid AAVP vectors encasing an Adenovirus genome in a filamentous phage shell for targeted gene therapy [49]. Yet, to the best of our knowledge the ability of phage virions to penetrate cancer spheroids has never been reported to date. The capacity of phages to cross or interact with eukaryotic tissues is not unanticipated, though. Phages are among the most abundant biological entities within our bodies, and have been found in blood, lymph, and other organs that have conventionally been considered sterile, including the brain [50]. M13 in particular, because of its filamentous structure, appears to possess the unique capability of bypassing anatomical and physiological barriers characterized by varying degrees of selectivity and permeability, including barriers that are commonly banned for much smaller therapeutic molecules or other phages [17]. A typical example is represented by the blood–brain barrier (BBB) as M13 can accumulate in the hippocampus and olfactory bulb of BALB/C mice shortly after intranasal administration [51]. Rapid and directional transcytosis of diverse bacteriophages across confluent cell layers originating from the gut, lung, liver, kidney, and brain have been reported, with the transport sporting a preferential apical-to-basolateral directionality thus providing a mechanistic explanation for the occurrence of phages within the body [16, 52]. Trafficking has been suggested to transit across the Golgi apparatus via the endomembrane system, which could tentatively explain the intracellular generation of ROS upon incubation with M13_CC_-RB and irradiation. These features are very convenient for photodynamic approaches since the disruptive activity of ROS is constrained to the immediate surroundings of the excited sensitizers. In particular, the high reactivity of ROS induced by PDT restricts their radius of action to 5–20 nm from the site of generation [53]. Accordingly, the observed spheroid collapse suggests that the engineered M13 phages are capable of vehiculating significant amounts of RB sensitizer deep into the spheroid core. Also recalling their high PS payload, tumor spheroid penetrating phage vectors may therefore represent a significant step forward for targeted PDT strategies.

In this scenario it is not surprising that a higher concentration of M13_CC_-RB is required to achieve a marked cytotoxic effect on the spheroid than that used on cells cultured in monolayer. The different behavior of 2D cells compared to 3D depends on their different physiological and physical properties that make 2D cells more susceptible to the cytotoxic action of drugs [54]. Indeed, the distance from the surface of the spheroid determines different gradients of oxygen tension, nutrients, and catabolites, resulting important in term of response to chemotherapy [27].

It is well recognized that PDT can lead to at least three forms of cell death, apoptosis, necrosis and/or autophagy [4]. We recently demonstrated that Ce6-conjugated M13 retargeted to EGFR receptor induces autophagic cell death in ovarian cancer cells [21]. Unlike the results observed for ovarian cancer cells, in the present paper we demonstrated that M13_CC_-RB mainly induced non-programmed cell death events in CC cells. The extent of damage, thus, the triggered mechanisms of cell death induced by PDT are influenced not only by the cell type but also by the PS, its cellular localization, the condition of light exposure (time and fluence rate) and O_2_ availability [55]. Photosensitizers which localize in the mitochondria usually stimulate apoptosis, whereas photosensitizers that localize in the plasma membrane mostly induce necrosis upon light exposure [35]. We recorded a significant increase in cells with damaged cell membrane, proportional to the concentration of RB and possibly dependent on the localization of the retargeted bacteriophage complex on the surface of the cells. Indeed, the amount of PS is usually responsible for the fast destruction of cell integrity and, thus, of proteins involved in the regulation of apoptosis or autophagy [4], bypassing these mechanisms of cell deaths, and resulting in extensive cell damage. ROS generated by PDT are the main responsible for direct damage of tumor cells because they rapidly oxidate the subcellular organelles [56]. Our results clearly show that M13_CC_-RB after irradiation generates a significant amount of intracellular ROS, in a PS concentration-dependent fashion. These ROS are conceivably responsible for the observed tumor cell death, as supported by the results we obtained with antioxidants. In PDT, ROS are generated according to two mechanisms of photoreaction: type I and type II. In type I the triplet excited state PS reacts directly with biomolecules, acquiring a hydrogen atom or an electron, to form radicals that react with water and molecular oxygen generating different radical oxygen species; in type II mechanism, energy is transferred from triplet excited state PS to molecular oxygen, forming the highly reactive singlet oxygen molecule [57]. The light-induced ROS generation by M13_CC_-RB was clearly demonstrated in a cell-free system comparing the energy *versus* electron transfer processes, using AR and ABMDMA assays, respectively. Upon binding the bacteriophage, RB significantly enhances type I mechanism (electron transfer process) for ROS generation, over type II mechanism (energy transfer process). The first mechanism is commonly favored in polar media but requires electron donating species. As previously shown by our group, due to the presence of electron-rich residues, both protein-RB [30] and phage-RB bioconjugates [22] can directly produce peroxides, without further addition of electron donating species. In general, this behavior suggests that the photodynamic performance of the PS not only depends on its molecular structure but can be significantly affected by the vector.

In conclusion, our study demonstrates the efficacy of anticancer photodynamic treatment using bacteriophages engineered to target CC cells. Despite their significant size, M13_CC_-RB phage bioconjugates display outstanding performances as targeted vector for PDT, deeply penetrating multicellular spheroids, and inducing light-dependent cytotoxic effects at picomolar concentrations. Spheroids represent a rapid screening method to assess the efficacy of PDT approaches, reducing the need of the highly relevant but low-throughput in vivo experiments for the preliminary assessment of PS uptake and penetration [58]. In general, the doses of phage at which lethal sensitization of CC cells could be observed (10^9–10^11 phages) is well compatible with phage intratumoral and intravenous (IV) injection in mouse tumor models [59], as well as for human administrations. For example in recent successful phage therapy trials, multiple daily doses of 10^9–10^10 phages were administered IV to a patient suffering from an antibiotic-resistant mycobacterial infection [60]. Likewise, the irradiation conditions are promptly translatable to clinical settings as the irradiance needed to activate the phage bioconjugate can be readily reached by therapeutic lasers, trimming down the irradiation times useful for sensitizer activation to few minutes, also in vivo [9]. Moreover, the use of specific sensitizers excitable in the near-infrared (NIR) window could allow for deeper tissue penetration of the light, enabling the treatment of malignancies deeper in the body. One limitation could be represented by the immunogenicity of the phage bioconjugates, which could hinder the efficacy in repeated applications. However, it is known that phages can also be (genetically or chemically) modified to increase their persistence in circulation and escape recognition by the complement system [61]. As such, our results pave the way for in vivo studies aimed at validating targetable and photoactivable phage nanovectors as adjuvant approaches to fight CC and other malignancies.

### Supplementary Information

Below is the link to the electronic supplementary material.

Supplementary file1 (DOCX 1094 KB)

## Data Availability

The datasets and materials generated and analysed during the current study are available from the corresponding authors on request.

## Abbreviations

7-AAD: 7-Aminoactinomycin
ABMDMA: 9,10-Anthracenediyl-bis(methylene) dimalonic acid
CC: Colorectal cancer
CDS: Coding sequence
DAMPs: Damage associated molecular patterns
EDC: 1-Ethyl-3-(3-dimethylaminopropyl) carbodiimide
EGFR: Epidermal growth factor receptor
FBS: Fetal bovine serum
FDA: Food and Drug Administration
FNR: Fibronectin Receptor
FN: Fibronectin
HRP: Horseradish peroxidase
IARC: International Agency for Research on Cancer
IPTG: Isopropyl β-D-1-thiogalactopyranoside
MFI: Mean fluorescence intensity
MMR: Mismatch repair
MTT: 3-(4,5-Dimethylthiazol-2-yl)-2,5-diphenyltetrazolium bromide
NAC: *N*-acetyl-l-cysteine
NHS: *N*-hydroxysuccinimide
NT: Untreated cells
PBS: Phosphate buffered saline
PDT: Photodynamic therapy
PE: Phycoerythrin
PEG: Polyethylene glycol
PFA: Paraformaldehyde
PS: Photosensitizer
PVDF: Polyvinylidene difluoride
RB: Rose Bengal
ROS: Reactive oxygen species
WS: Working solution
