# Supplementary material for: Molecular engineering of a spheroid-penetrating phage nanovector for photodynamic treatment of colon cancer cells
Source: Cell Mol Life Sci. 2024 Mar 17;81(1):144. doi: 10.1007/s00018-024-05174-7 (PMC10944812; doi:10.1007/s00018-024-05174-7)
Supplement: Supplementary file 1 — Supplementary file1 (DOCX 1094 KB) [file 18_2024_5174_MOESM1_ESM.docx]

**Molecular engineering of a spheroid-penetrating phage nanovector for photodynamic treatment of colon cancer cells**

Eleonora Turrini^1,^*, Luca Ulfo^2,^*, Paolo Emidio Costantini^2,^*, Roberto Saporetti^3^, Matteo Di Giosia^3^, Michela Nigro^2^, Annapaola Petrosino^2^, Lucia Pappagallo^2^, Alena Kaltenbrunner^2^, Andrea Cantelli^3,4^, Valentina Pellicioni^1^, Elena Catanzaro^5,6^, Carmela Fimognari^1^, Matteo Calvaresi^3,7,§^ and Alberto Danielli^2,7,§^

*^1^ Dipartimento di Scienze per la Qualità della Vita (QUVI) - Alma Mater Studiorum - Università di Bologna, C.so D’Augusto, 237, 47921 Rimini, Italy*

*^2^ Dipartimento di Farmacia e Biotecnologie (FaBiT) – Alma Mater Studiorum - Università di Bologna, Via Francesco Selmi 3, 40126 Bologna, Italy*

*^3^ Dipartimento di Chimica “Giacomo Ciamician” – Alma Mater Studiorum - Università di Bologna, Via Francesco Selmi 2, 40126 Bologna, Italy*

*^4^ CNR Institute of Molecular Genetics "Luigi Luca Cavalli-Sforza" Unit of Bologna, Italy.*

*^5^ Cell Death Investigation and Therapy (CDIT) Laboratory, Department of Human Structure and Repair, Ghent University, Corneel Heymanslaan 10, 9000 Ghent, Belgium*

*^6^ Cancer Research Institute Ghent (CRIG), Ghent, Belgium*

*^7.^Health Sciences and Technologies - Interdepartmental Center for Industrial Research (CIRI-SDV), University of Bologna, Italy*

* ET, LU and PEC contributed equally to this work

^§^ to whom correspondence should be addressed: [alberto.danielli@unibo.it](mailto:alberto.danielli@unibo.it), [matteo.calvaresi3@unibo.it](mailto:matteo.calvaresi3@unibo.it)

**Supplementary Information (SI)**

**Supplementary Figure S1.** The M13_CC_ phage shows a specific tropism towards the CC HT29 cell line.


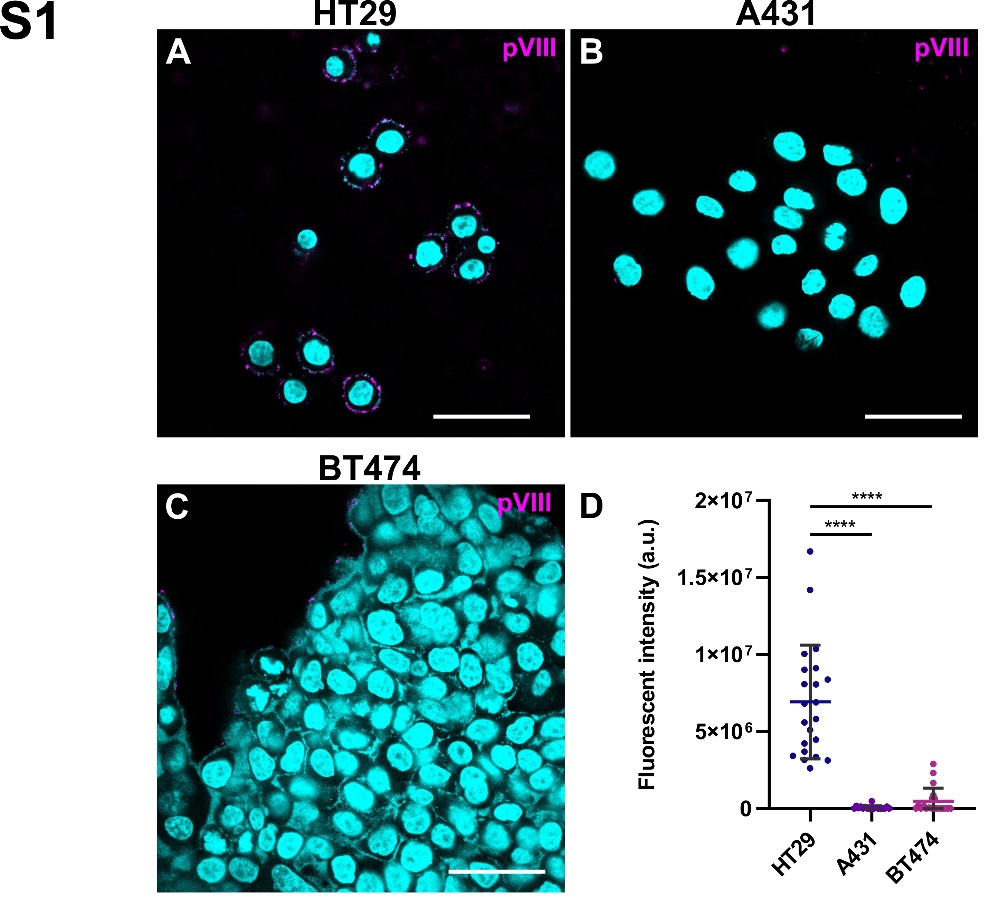


***Supplementary Fig. 1* -** Immunohistochemical confocal microscopy of (A) HT29, (B) A431 and (C) BT474 cell lines incubated with M13_CC_. Nuclei, stained by Hoechest, are in cyan while the major coating protein pVIII of the phage is in magenta. Scale bar= 50 µm. (D) Quantitative analysis performed on confocal images (twenty regions of interest for each cell line, n=20). Statistical significance was calculated by one-way parametric ANOVA in comparison to the control (PBS). ****p < 0.0001.

**Supplementary Figure S2.** Lethal photosensitization of CC lines by the M13_CC_-RB phage bioconjugate.


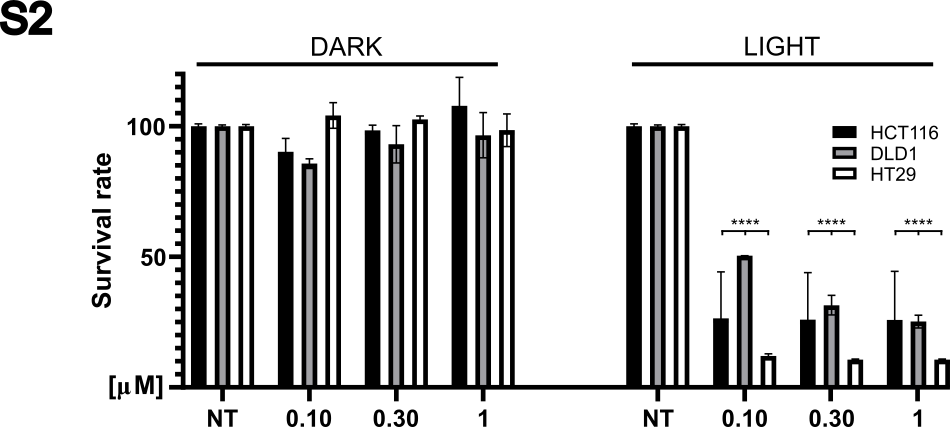


**Supplementary Fig. 2 –** Cytotoxic effects of M13_CC_-RB on HCT116, DLD1, and HT29 CC cell lines kept in the dark or irradiated for 30 min, after 24 h from treatment analysed through MTT assay. Cells were incubated with increasing concentrations of M13_CC_-RB conjugated phages (0.10, 0.30, and 1.00 [µM]). 'NT' indicates non-treated cells. All experiments were performed according to the protocols detailed in the Materials and Methods section. Error bars represent the standard deviation from triplicate experiments (n=3). Statistical significance was calculated by two-way ANOVA in comparison to the control (NT). ****p < 0.0001.

**Supplementary Figure S3.** Photodynamic cell death mechanisms triggered by the M13_CC_-RB vector following 10 or 30 min irradiation in DLD1 and HT29 CC cell lines.


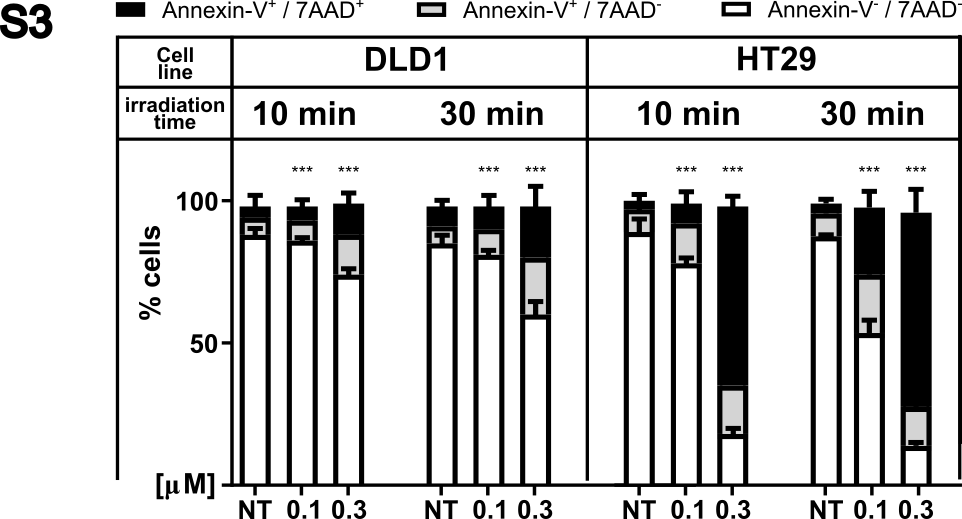


**Supplementary Fig. 3 -** Analysis of cell death mechanisms in DLD1 and HT29 CC cell lines following photodynamic treatment. The cells were treated with M13_CC_-RB conjugated phages at concentrations of 0.1 and 0.3 µM and then subjected to 10 or 30 minutes of irradiation. Cell death was quantified by staining with Annexin-V and 7AAD, where Annexin-V+/7AAD- indicates early apoptosis, Annexin-V+/7AAD+ indicates late apoptosis/necrosis, and Annexin-V-/7AAD- indicates living cells. 'NT' denotes the non-treated cells. The percentage of cells in each category is shown, with error bars representing standard deviation from triplicate experiments (n=3). The experiments were carried out in accordance with the Materials and Methods section. Statistical significance was calculated by two-way ANOVA in comparison to the control (NT). ****p < 0.0001.

**Supplementary Figure S4.** Lack of caspase 3/7 activation after photodynamic treatment with M13_CC_-RB phage bioconjugates.


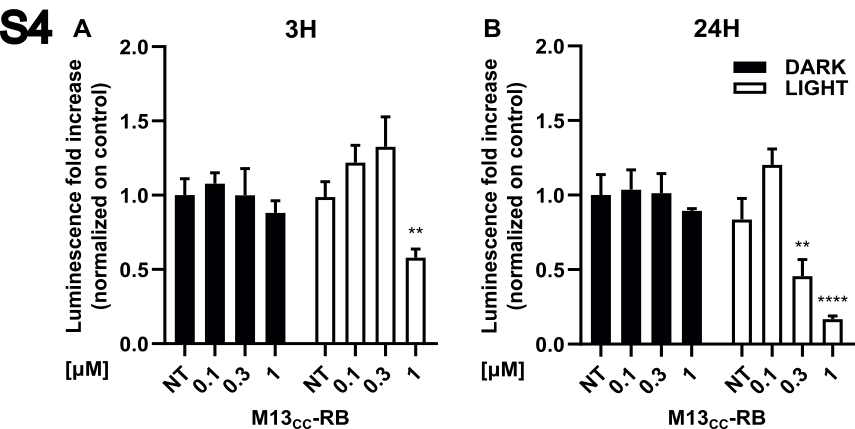


***Supplementary Fig. 4* -** Caspase-Glo® 3/7 assay results depicting caspase activation after photodynamic treatment with M13_CC_-RB conjugated phages in dark and light conditions on HT29 cell line. (A) Shows luminescence fold increase normalized to control at 3 h post-treatment across three different phage concentrations (0.1, 0.3, and 1 µM). (B) Luminescence at 24 h post-treatment. Black bars denote dark conditions; white bars denote light conditions. Results are mean ± standard deviation of three biological independent replicates (n=3), illustrating the temporal and dose-dependent effects of the treatment on apoptosis. Statistical significance was calculated by one-way parametric ANOVA in comparison to the control (NT – DARK).

**Supplementary Figure S5.** Flow cytometry analysis to evaluate the morphology of CC cells treated with M13_CC_-RB.


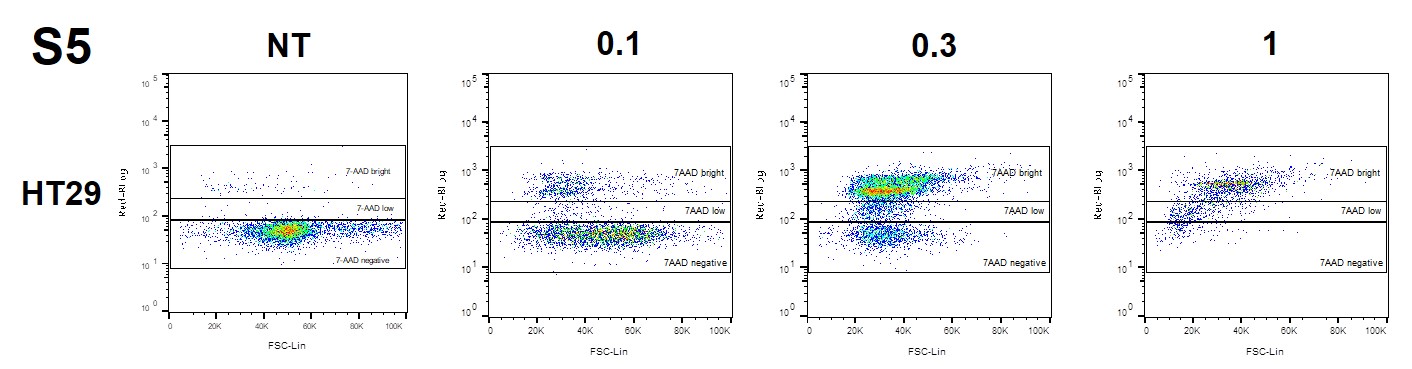


***Supplementary Fig. 5*** - Flow cytometry analysis to evaluate the morphology of CC cells treated with M13_CC_-RB. The cells were exposed to increasing concentrations of M13_CC_-RB conjugated phages (0, 0.1, 0.3, and 1 [µM]), as indicated. The dot plots display the forward light scatter (FSC) versus the 7-AAD fluorescence. This permits a discrimination of viable cells which exclude the dye totally (7-AAD negative), from apoptotic cells that fluoresce dimly (7-AAD low), and necrotic and late apoptotic cells that fluoresce brightly (7-AAD bright) and are characterized by a decrease in FSC.

**Supplementary Figure S6.** Confocal microscopy analysis of M13_CC_-RB phage penetration in transparized CC spheroids.


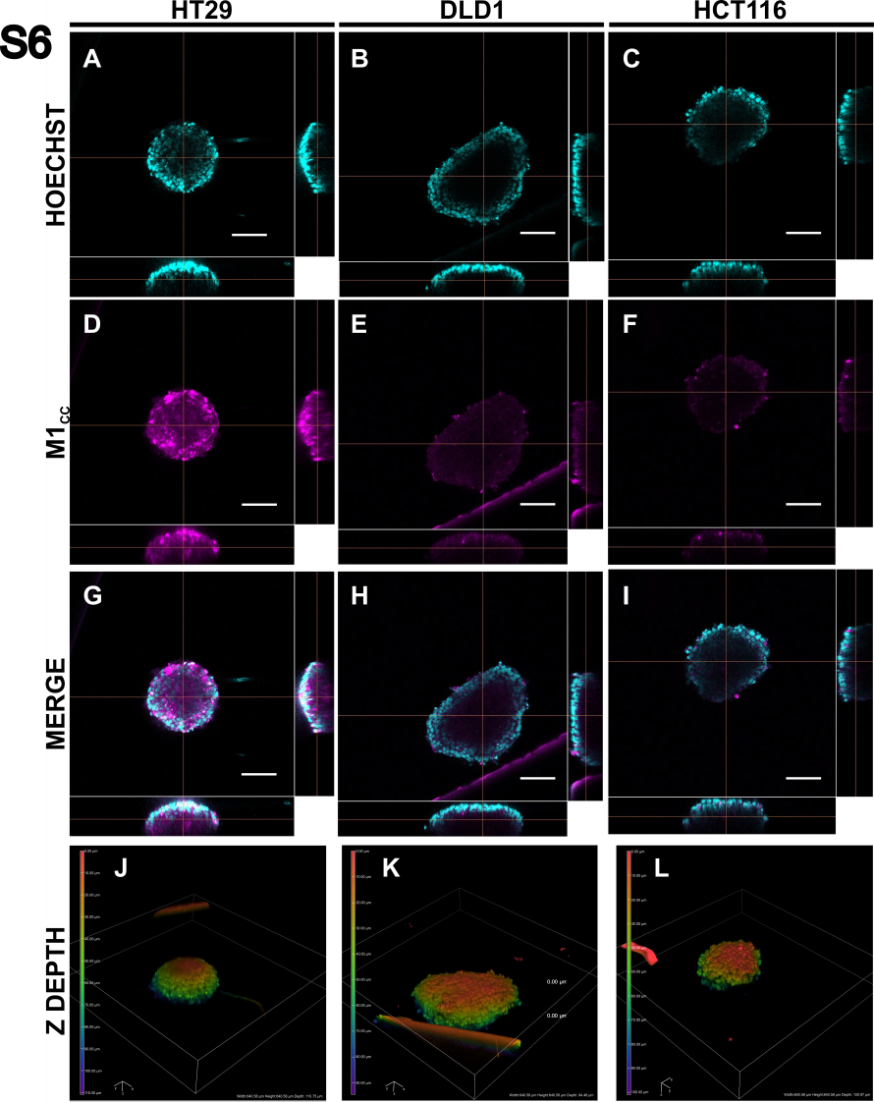


**Supplementary Fig. 6 -** Confocal microscopy analysis of M13_CC_-RB conjugated phage penetration in CC spheroids. Spheroids from HT29 (A, D, G, J), DLD1 (B, E, H, K), and HCT116 (C, F, I, L) cell lines were incubated with phages and simultaneously stained with Hoechst for nuclear localization. Panels A-C show Hoechst staining (cyan), panels D-F show phage distribution (magenta), and panels G-I are the merged images, displaying the localization of the phages in relation to the nuclei. Panels J-L depict the Z-depth reconstructions for a 3D view of the phages in the spheroids. All images were captured using the same confocal microscope settings, with a constant 3 µM concentration of RB equivalents. The spheroids were cleared with glycerol for imaging transparency. Scale bars correspond to 100 µM.
